# Diagnosis and treatment of cryptorchid testicular torsion in children: A 12-year retrospective study

**DOI:** 10.3389/fped.2022.968997

**Published:** 2022-08-22

**Authors:** Pengyu Chen, Zhilin Yang, Nana Chen, Lei Liu, Jiahong Su, Mengkui Sun, Shoulin Li

**Affiliations:** ^1^Department of Urology, Shenzhen Children's Hospital, China Medical University, Shenzhen, China; ^2^Department of Urology, Shenzhen Children's Hospital, Shenzhen, China

**Keywords:** children, cryptorchidism, diagnosis, orchiectomy, testicular torsion, treatment

## Abstract

**Purpose:**

To investigate the clinical characteristics, treatment, and prognosis of cryptorchid testicular torsion in children.

**Methods:**

The clinical data of 25 children who received treatment for cryptorchid testicular torsion between January 2010 and December 2021 were retrospectively reviewed. The median age of the patients was 64.5 months (range: 2 months to 15 years). All patients had unilateral torsion, and the duration of symptoms ranged from 3 to 192 h.

**Results:**

Among the 25 patients, five underwent orchidopexy, while the remaining 20 underwent orchiectomy. After 6 months to 8 years of follow up, the 20 patients who had undergone orchiectomy had a well-developed testis on the healthy side. Four of the five patients who had undergone orchidopexy of the affected testis had well-developed testes bilaterally, while one experienced testicular atrophy.

**Conclusion:**

Cryptorchid testicular torsion is a rare urological emergency that displays a delayed presentation and is often misdiagnosed. Clinicians need to carefully review the patient's medical history and ultrasound findings and perform a thorough physical examination to make a correct diagnosis. Active testicular exploration is required for patients suspected to have cryptorchid testicular torsion, and the decision to perform orchidopexy or orchiectomy depends on the intraoperative situation.

## Introduction

Cryptorchidism or undescended testes is one of the most common congenital urinary diseases, characterized by the absence of testicles or inability to palpate the testicles in the scrotum (1). The incidence of cryptorchidism is ~1% (2). Testicular torsion is a common scrotal emergency in pediatric urology, with an incidence of 3.8 per 100,000 boys (3). Cryptorchid testicular torsion accounts for 5–15 % of cases of testicular torsion (4–6). Testicular torsion is caused by rotation of the testes and epididymis around the spermatic vessels, resulting in an acute interruption of testicular blood supply. If torsion is not be corrected by immediate surgical intervention, the possibility of testicular ischemic injury or even necrosis is greatly increased (7, 8). Thus, after the onset of testicular torsion related symptoms, the “prime time” for rescuing the testis is 4 to 8 h. The testicular salvage rate through surgical reduction within the first 6 h after the onset of symptoms is 90–100%, and it falls to 10% beyond 24 h (9).

Cryptorchid testicular torsion is very rare in children. It is often misdiagnosed due to the similarity in clinical characteristics between cryptorchid testicular torsion and other conditions such as incarcerated indirect inguinal hernia, inguinal lymphadenitis, and acute abdomen. Furthermore, limit by the diagnosis and treatment experience in grass roots hospitals, the window of opportunity for salvaging the testis is frequently missed (10). Thus, in this study, we aimed to investigate the clinical characteristics, treatment, and prognosis of cryptorchid testicular torsion in children to provide a reference for the clinical diagnosis and treatment of this disease.

## Materials and methods

### Patients

The clinical data of children with cryptorchid testicular torsion treated in the Department of Urology of Shenzhen Children's Hospital between January 2010 and December 2021 were retrospectively collected. Inclusion criteria were as follows: (1) intraoperatively confirmed torsion of the undescended testis, (2) follow-up for at least 6 months after surgery, and (3) complete medical records and data. Exclusion criteria were as follows: (1) no surgical treatment and (2) no neonatal testicular torsion. A total of 245 patients with testicular torsion were treated in our hospital in the past 12 years. Of these, 26 patients with incomplete medical records and follow-up data, 23 new-borns with testicular torsion, and 171 patients with intrascrotal testicular torsion were excluded; 25 patients with cryptorchid testicular torsion were finally included in this study.

Patients' clinical data were collected, including the age of onset, clinical symptoms, duration of symptoms (defined as the interval between the onset of symptoms and the start of surgery), concomitant diseases, operation-related data, laboratory and pathological examination results, ultrasound results, and postoperative follow-up data. This study was approved by the ethical committee of Shenzhen Children's Hospital (No. 2022014), and informed consent was obtained from the patients' legal guardians.

### Management and follow-up

**Figure 1:**
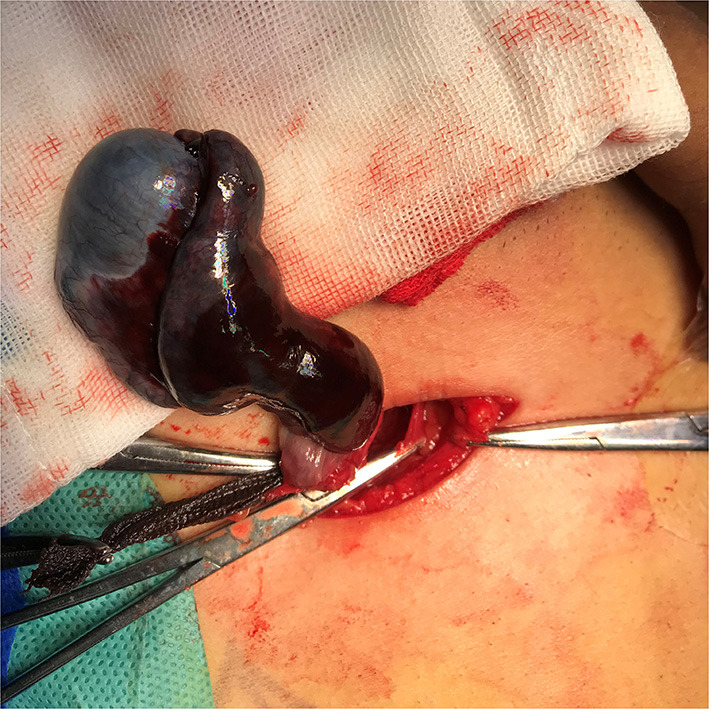
Inguinal exploration in a 12-year-old patient with left cryptorchidism reveals 720° torsion of the left testis and ischemic necrosis of the testis and epididymis.

All children were treated in the emergency department of our hospital. The green channel was opened to ensure that the patients underwent surgery within 1 h of admission. Surgery was performed by experienced urologists in our hospital. First, the child was placed in the supine position. After administering anesthesia, a transverse incision was made in the inguinal canal on the affected side. The external oblique aponeurosis was incised, and the spermatic vessels were freed downward. The testis was lifted out of the thecal capsule and quickly restored. Warm gauze was applied externally for 20 min, and restoration of testicular blood circulation was observed. If the color of the testis improved and fresh bleeding was observed after incising the tunica albuginea, orchiopexy was performed. If there was no fresh bleeding, the testis was considered necrotic and orchiectomy was performed with the consent of the parents (Figure 1). The contralateral testis was routinely fixed. The patients were followed up at 2 weeks, 2, 6 months, and 1 year postoperatively, and testicular recovery after testicular torsion was evaluated through physical and ultrasonic examination. The primary outcome was the occurrence of postoperative testicular atrophy.

### Statistical analysis

Data were analyzed using IBM SPSS 22.0 (Chicago, IL, USA). Categorical variables are presented as number of cases and percentages. Non-normally distributed continuous variables are presented as median with interquartile range.

## Results

A total of 25 patients with cryptorchid testicular torsion were included in this study, accounting for 10.2% (25/245) of the total number of patients with testicular torsion during the same period. The age of the patients ranged from 2 months to 15 years, with a median age of 64.5 months. The left testis was affected in 18 patients and the right in sevenThe duration of symptoms ranged from 3 to 192 h, with a median time of 25 h. Most patients experienced swelling or pain in the inguinal region, while a few experienced symptoms, such as abdominal pain, vomiting, and restlessness. All patients underwent preoperative ultrasonography, with 23 of them showing incomplete testicular descent with torsion. The diagnostic accuracy of ultrasound was 92.0% (23/25).

After admission, blood samples were collected for routine blood examination. The mean platelet volume (MPV) of the 25 patients with cryptorchid testicular torsion ranged between 8.1 and 12.3 fL. Regarding surgical treatment, five patients underwent orchiopexy while the remaining 20 underwent orchiectomy. Various degrees of testicular torsion (range: 90to 1,260°, median: 540°) were found during surgery. Based on the results of preoperative examination, four patients were thought to have bilateral cryptorchidism torsion; however, during surgery, they displayed unilateral torsion. Three patients had cerebral palsy and one patient was diagnosed with 46XY disorder of sex development and had hypospadias.

All 25 patients were followed up for a range of 6 months to 8 years. In the 20 patients who underwent orchiectomy, the contralateral testicles were in the normal position, and ultrasound showed good testicular blood supply. Among the five patients who underwent orchiopexy, four had normal testis while one developed testicular atrophy. This patient had poor testicular blood circulation during surgery, but the parents insisted on preserving the testis, whichatrophied 2 months later. Ultrasonography showed that the testicular volume was significantly reduced and that there was no blood flow in the testis. The success rate of testicular rescue was 16.0% (4/25). The results are summarized in Table 1.

**Table 1:** Baseline characteristics of patients.

**Parameters**	**Value**
Age (month)	64.5 (10.1–106.3)
infantile (29 days−1 year)	7
early childhood (1−3 years)	4
preschool (3–6 years)	4
school age (6–10 years)	6
puberty (10–17 years)	4
**Affected side**, ***n*** **(%)**	
Left	18 (72.0)
Right	7 (28.0)
Duration of symptoms (h)	25 (22–72)
**Presenting symptoms**	
Inguinal mass and/or Inguinal pain	21
Abdominal pain	4
Restless	5
Limping	1
Vomiting	6
**Surgery**, ***n*** **(%)**	
Orchidopexy	5 (20.0)
Orchiectomy	20 (80.0)
Torsion angle (°)	540 (360–720)
Surgery time (min)	62.5 (55.0–82.5)
WBC (10^9^/L)	11.9 (9.6–16.2)
Neutrophil (10^9^/L)	7.1 (4.3–9.3)
Lymphocyte (10^9^/L)	3.9 (2.2–6.2)
MPV (fL)	9.8 (9.2–10.4)
NLR	1.8 (0.9–3.7)
**Follow-up of children with orchidopexy**	
Follow-up time (month)	18 (12–25)
Good blood supply	4
No blood supply and atrophy	1
Ipsilateral testicular salvage, *n* (%)	4 (16.0)

WBC, white blood cell; MPV, mean platelet volume; NLR, neutrophil-lymphocyte ratio.

## Discussion

Cryptorchid testicular torsion is rare in children, with only a few reports in the literature. This study summarizes the clinical data of 25 patients with cryptorchid testicular torsion to provide a reference for the clinical diagnosis and treatment of this disease. The main findings of our study were as follows: (1) 10.2% of the patients with testicular torsion had cryptorchid testicular torsion occurring in the inguinal canal, (2) initial symptoms such as abdominal pain and vomiting may indicate cryptorchid testicular torsion, (3) ultrasound has a diagnostic accuracy of 92.0% and may be useful as a diagnostic tool for cryptorchid testicular torsion, (4) MPV may be a valuable predictive marker for cryptorchid testicular torsion, (5) surgical treatment options for cryptorchid testicular torsion include orchiopexy and orchiectomy, depending on the intraoperative situation, and (6) contralateral testis fixation following surgical treatment showed satisfactory results and could be routinely performed.

Although cryptorchid testicular torsion is relatively rare, the incidence of testicular torsion in children with cryptorchidism is 10 times higher than that in normal children (11). Cryptorchid testicular torsion accounts for 5–15% of cases of testicular torsion (4–6), similar to the present study where 10.2% of patients with testicular torsion had cryptorchid testicular torsion. Williamson et al. (11) found the incidence of cryptorchid testicular torsion in patients with testicular torsion to be 6.8% (20/293), while Zhong et al. (5) found it to be 13.0% (24/185).

Testicular torsion of cryptorchidism can occur at all ages (5). Cases of cryptorchid testicular torsion have been reported in patients ranging in age from 16 days to 14 years (6, 12, 13). In our study, the age of onset was mostly before puberty, specifically during infancy and early childhood. More prepubertal children were diagnosed with cryptorchidism than adolescents. This may be due to the increased awareness of testicular abnormalities, such as cryptorchidism, in children and their parents and the increasing availability of treatment options.

The pathogenesis of cryptorchid testicular torsion is still unclear. Cryptorchid testicular torsion may be related to abnormal spasm or contraction of the levator muscle, which is more prevalent in children with cerebral palsy or neuromuscular diseases (14). A recent study reported that 11 of 60 patients with cryptorchid testicular torsion had neurological diseases (15). In our study, cryptorchid testicular torsion was complicated by cerebral palsy in three patients. However, at present, cryptorchid testicular torsion complicated by cerebral palsy or neuromuscular diseases has only been described in case reports, and the correlation between these disease is unknown (14). Some scholars believe that a space-occupying lesion in the testis could lead to spermatic cord distortion or changes in the position of the testis, which could cause testicular torsion. Among the 19 cases of cryptorchid testicular torsion reported by Yang et al. (6) two were diagnosed as cryptorchid testicular torsion with testicular teratoma. Additionally, the lack of anatomical fixation in the scrotum and unclosed sheath process in patients with cryptorchidism can increase the degree of freedom of testicular rotation and lead to testicular torsion (16). If the testicular mesentery is absent too long, or inadequately fixed, there is a defect of attachment between the testes and external spermatic fascia, which will increase testicular activity and induce testicular torsion (17, 18).

In cryptorchidism, the affected testis is located either at the entrance of the scrotum, superficial inguinal layer, inguinal canal, or abdominal cavity, while cryptorchid testicular torsion occurs mostly in the inguinal canal. Among the 19 cases of cryptorchid testicular torsion reported by Yang et al. (6) 18 occurred in the inguinal canal, while one case in the abdominal cavity. In our study, cryptorchid testicular torsion occurred in the inguinal canal in all 25 patients. Compared to the high incidence of affected right testis in children with cryptorchidism, cryptorchid testicular torsion occurs more frequently on the left side, which may be related to the fact that the spermatic cord is longer on the left side (17). In studies conducted by Zhong et al. (5) and Sener et al. (13) 68.5% (24/35) of cases of cryptorchid testicular torsion occurred on the left side.

The most common clinical manifestation of cryptorchid testicular torsion is swelling or pain in the inguinal region with emptiness in the ipsilateral scrotum, and infants often experience restlessness and reduced intake. Some older children experience symptoms, such as vomiting, abdominal pain, and lameness, which may be due to the torsion of spermatic vessels, stimulating the peritoneum and causing abdominal nerve reflex (19). Pogorelic et al. (20) reported that 90.9% (10/11) of patients with cryptorchidism torsion experienced abdominal pain. In this study, eight children experienced abdominal pain or vomiting, and intraoperative exploration showed testicular necrosis for which orchiectomy was performed. For children presenting with abdominal pain or vomiting as the initial symptom, a comprehensive physical examination should be conducted to avoid adverse consequences caused by delayed or misdiagnosis.

Doppler ultrasound combined with clinical signs is often used to diagnose or exclude testicular torsion. The following can be observed on Doppler ultrasound: (1) twisting and thickening of the spermatic cord on the affected side, (2) enlargement of the affected testis, (3) unevenness of parenchymal echo, (4) weak or no blood flow signal in the testis, and (5) increased blood flow in the tissue near the testis (21). In the first 4 h of testicular torsion, the testicular artery is not damaged and the testis may still be healthy, but as time progresses, testicular focal hemorrhage and necrosis gradually worsens, and abnormal echoes are observed in the testicular parenchyma. Color Doppler ultrasonography can be used to diagnose testicular torsion with a sensitivity of 100% and specificity of 76% (22). In our study, ultrasound had a diagnostic accuracy of 92.0%. It is less accurate in evaluating cryptorchid testicular torsion due to the interference of surrounding tissue and intestinal gas (23). If ultrasound results are unclear and the degree of suspicion of testicular torsion is low, computed tomography or magnetic resonance imaging examination can be considered (24, 25).

Aside from imaging examination, hematological parameters have predictive value in the differential diagnosis of testicular torsion. White blood cell count and neutrophil-lymphocyte ratio are higher than normal in patients with testicular torsion but lower in those with epididymal orchitis (26). Larger platelets are more active in enzyme activity and metabolism; thus, MPV can be regarded as an indirect marker of platelet function (27). Cicek et al. (28) reported that the cut-off value of MPV for predicting testicular torsion was 7.7 fL, with a sensitivity of 62% and a specificity of 96%. In our study, MPV of the 25 patients with cryptorchid testicular torsion ranged between 8.1 and 12.3 fL, which was consistent with the results reported by Cicek et al., however, the optimal cut-off value for MPV needs further verification.

If patients with cryptorchid testicular torsion who are misdiagnosed with incarcerated indirect inguinal hernia are treated rashly with incarcerated hernia reduction, the degree of their testicular injury will worsen, resulting in adverse consequences. For patients who have an impalpable testis in the scrotum without induced abdominal or inguinal pain, cryptorchid testicular torsion must be considered as a differential diagnosis, and examinations of the abdomen, inguinal region, and genitourinary system should be improved as soon as possible. Bayne et al. (29) showed that delay or misdiagnosis of testicular torsion is often associated with isolated abdominal pain, testicular dysplasia, or recent genital trauma.

Due to uncertainties in the diagnosis of cryptorchid testicular torsion, the window of opportunity to rescue the testis is often missed; therefore, it is critical to detect and treat undescended testis early (30). Studies have shown that spontaneous descent of the testis in children with cryptorchidism occurs very rarely after 6 months of age. The purpose of surgical treatment for cryptorchidism is to fix the testis in the relatively cold scrotum to reduce the incidence of infertility and allow for the early detection of testicular tumors (31). Among the 25 patients in the present study, 10 were examined and diagnosed with cryptorchidism before testicular torsion; however, because the parents were unaware of the clinical complications of cryptorchidism, no immediate surgery was performed. The parents of six patients noticed scrotal emptiness but did not insist on any treatment. The parents of nine other patients did not examine their children's scrotum before the onset of the disease. Therefore, it is necessary to perform a genital examination after birth and educate parents on cryptorchidism, so that complete testicular fixation can be performed immediately to avoid complications caused by testicular torsion.

Our hospital opens a green channel for patients with testicular torsion in the emergency department, which greatly reduces the time of hospitalization and preoperative preparation. Another key factor for the success rate of testicular rescue is the degree of testicular torsion (32). If the testicular torsion is >360°, it can cause testicular atrophy within 4 h, and if it is <360°, the testis can be rescued within 24 h (33). In one of our patients, the torsion time was 3 h, and the degree of torsion was 360°. During surgery, the color of the testis was black, and there was no fresh blood flow after the tunica albuginea was incised. Given that the testis was necrotic, orchiectomy was performed.

With the advancement in laparoscopic technology, laparoscopic orchiopexy has become the treatment of choice for high cryptorchidism (34). Laparoscopic exploration can be performed in children with suspected intra-abdominal testicular torsion, but given that the intra-abdominal testis has a higher risk of becoming cancerous, experts recommend that such torsional testicles be removed (35). However, for patients with inguinal cryptorchid testicular torsion, we recommend an open inguinal approach and rapid reduction after testicular torsion is found. If testicular blood flow is restored, orchiopexy can be performed. If there is no obvious change in testicular color, the tunica albuginea should be incised to observe section bleeding, and if the oozing of blood is observed within 10 min, testicular retention can be considered. If there is no outflow of fresh blood and testicular necrosis is observed, orchiectomy is recommended. Zhao et al. (3) reported that 41.9% of children with testicular torsion underwent orchiectomy, while Yang et al. (6) reported that 78.9% of the patients with cryptorchid testicular torsion underwent orchiectomy. In our study, orchiectomy was performed for 80.0% (20/25) of cryptorchid testicular torsion cases, which was higher than that performed for testicular torsion in the scrotum.

Routine contralateral testicular preventive fixation for patients with cryptorchid testicular torsion is still controversial. At present, there is no report on the long-term follow-up results of contralateral testis after cryptorchid torsion. However, given the higher testicular resection rate of cryptorchid testicular torsion, contralateral testicular fixation is necessary to ensure normal reproductive function of the contralateral testis (5, 36). Among the urology group members of the American Academy of Pediatrics, 79.3 and 81.1% recommended contralateral testicular fixation in patients with preadolescent and post-adolescent testicular torsion, respectively (37). In our case, the contralateral testis was routinely fixed, and postoperative follow-up showed that the contralateral testis developed well with no signs of torsion.

Children need to be followed up for a long time after testicular torsion, and the testicular development is evaluated by ultrasound. Studies have reported that 38.94–41.4% of patients with testicular torsion who had undergone testicular fixation on the affected side eventually developed testicular atrophy (38, 39). Yang et al. (6) reported that among five patients with cryptorchid testicular torsion in whom the affected testis was retained, only one experienced postoperative ischemic atrophy, and the rest of the patients had normal testicular blood flow during follow-up. In our study, among the five patients in whom the affected testis was preserved, one had testicular necrosis during the surgery, but his parents requested that the testis be preserved; testicular atrophy occurred 2 months postoperatively in him. However, due to the small sample size, we cannot conclude on whether cryptorchid testicular torsion has a better prognosis than testicular torsion in the scrotum.

Our study has some limitations. First, the surgeries were performed by different urologists, which may have impacted our results. Second, our follow-up period was relatively short, and there might have been errors in assessing long-term prognosis.

## Conclusion

Cryptorchid testicular torsion is a rare urological emergency that is often misdiagnosed. Clinicians should conduct a comprehensive physical examination and carefully review the patient's medical history and ultrasound results to confirm the diagnosis. If cryptorchid testicular torsion is suspected, emergency surgical exploration is needed, and orchiopexy or orchiectomy should be performed depending on the intraoperative situation. For children in whom the affected testis is retained, long-term follow-up is needed to observe for the occurrence of testicular atrophy or malignant transformation.

## Data availability statement

The raw data supporting the conclusions of this article will be made available by the authors, without undue reservation.

## Ethics statement

The studies involving human participants were reviewed and approved by Medical Research Ethics Committee of Shenzhen Children's Hospital. Written informed consent to participate in this study was provided by the participants' legal guardian/next of kin.

## Author contributions

Conceptualization and visualization: PC and SL. Data curation: PC and MS. Formal analysis: NC and LL. Funding acquisition: MS and SL. Investigation: JS and ZY. Methodology: ZY and NC. Supervision: LL and JS. Writing–original draft: PC. All authors writing–review and editing, contributed to the article, and approved the submitted version.

## Funding

This study was supported by grants from Shenzhen Fund for Guangdong Provincial High-level Clinical Key specialties (Grant No: SZXK035) and National Natural Science Foundation of China (Grant No: U1904208).

## Conflict of interest

The authors declare that the research was conducted in the absence of any commercial or financial relationships that could be construed as a potential conflict of interest.

## Publisher's note

All claims expressed in this article are solely those of the authors and do not necessarily represent those of their affiliated organizations, or those of the publisher, the editors and the reviewers. Any product that may be evaluated in this article, or claim that may be made by its manufacturer, is not guaranteed or endorsed by the publisher.

## Abbreviations

MPV: Mean platelet volume.
